# Two Host Factors Regulate Persistence of H7^a^-Specific T Cells Injected in Tumor-Bearing Mice

**DOI:** 10.1371/journal.pone.0004116

**Published:** 2009-01-07

**Authors:** Marie-Christine Meunier, Chantal Baron, Claude Perreault

**Affiliations:** 1 Institute for Research in Immunology and Cancer, University of Montreal, Montreal, Quebec, Canada; 2 Department of Medicine, University of Montreal, Montreal, Quebec, Canada; 3 Division of Hematology, Maisonneuve-Rosemont Hospital, Montreal, Quebec, Canada; New York University School of Medicine, United States of America

## Abstract

**Background:**

Injection of CD8 T cells primed against immunodominant minor histocompatibility antigens (MiHA) such as H7^a^ can eradicate leukemia and solid tumors. To understand why MiHA-targeted T cells have such a potent antitumor effect it is essential to evaluate their *in vivo* behavior. In the present work, we therefore addressed two specific questions: what is the proliferative dynamics of H7^a^-specifc T cells in tumors, and do H7^a^-specific T cells persist long-term after adoptive transfer?

**Methodology/Principal Findings:**

By day 3 after adoptive transfer, we observed a selective infiltration of melanomas by anti-H7^a^ T cells. Over the next five days, anti-H7^a^ T cells expanded massively in the tumor but not in the spleen. Thus, by day 8 after injection, anti-H7^a^ T cells in the tumor had undergone more cell divisions than those in the spleen. These data strongly suggest that anti-H7^a^ T cells proliferate preferentially and extensively in the tumors. We also found that two host factors regulated long-term persistence of anti-H7^a^ memory T cells: thymic function and expression of H7^a^ by host cells. On day 100, anti-H7^a^ memory T cells were abundant in euthymic H7^a^-negative (B10.H7^b^) mice, present in low numbers in thymectomized H7^a^-positive (B10) hosts, and undetectable in euthymic H7^a^-positive recipients.

**Conclusions/Significance:**

Although in general the tumor environment is not propitious to T-cell invasion and expansion, the present work shows that this limitation may be overcome by adoptive transfer of primed CD8 T cells targeted to an immunodominant MiHA (here H7^a^). At least in some cases, prolonged persistence of adoptively transferred T cells may be valuable for prevention of late cancer relapse in adoptive hosts. Our findings therefore suggest that it may be advantageous to target MiHAs with a restricted tissue distribution in order to promote persistence of memory T cells and thereby minimize the risk of cancer recurrence.

## Introduction

Adoptive transfer of allogeneic T lymphocytes, used primarily for treatment of hematopoietic malignancies, has met with a remarkable success rate [1], [2]. Accordingly, the so-called graft-versus-leukemia (GVL) effect represents the most conclusive documentation that the immune system can cure cancer in humans [1], [3], [4]. The GVL effect is due mainly, and perhaps exclusively, to recognition of minor histocompatibility antigens (MiHAs) [1], [2], [5]. We reported that injection of CD8 T cells primed against the model immunodominant H7^a^ MiHA could eradicate not only leukemia but also melanoma in mouse [6], [7]. The chain of events leading to melanoma eradication by anti-H7^a^ T cells involves the following steps [7]. First, primed T cells accumulate at the tumor site. This initial step depends on interaction between Vla-4 on T cells and Vcam-1 on tumor blood vessels. Second, local release of IFN-γ by anti-H7^a^ T cells has two crucial effects: inhibition of tumor angiogenesis and upregulation of MHC I expression on tumor cells. Finally, anti-H7^a^ CD8 T cells undergo antigen-specific granule exocytosis in the tumor and thereby kill tumor cells. Of note, T cells specific for a single MiHA, such as H7^a^, never elicit graft-versus-host disease even when their target MiHA is ubiquitously expressed in recipient tissues and organs [6]–[8].

To understand why MiHA-targeted T cells are so effective we deemed it essential to evaluate their *in vivo* behavior. In the present work, we therefore addressed two specific questions. First, what is the proliferative dynamics of H7^a^-specifc T cells in the tumor? This is a critical issue since cancer refractoriness to immunotherapy is commonly due to failure of T cells to penetrate and accumulate in the tumors [9]–[12]. Second, do H7^a^-specific T cells persist long-term, and is protracted T-cell reactivity to H7^a^ necessary to prevent tumor recurrence? We report that the proliferation dynamics of anti-H7^a^ CD8 T cells is dramatically different in the tumor compared with the spleen. We present evidence that the massive accumulation of anti-H7^a^ in melanomas is largely due to extensive *in situ* proliferation. Moreover, we found that the long-term fate of H7^a^-specific T cells was dictated by two host factors: thymic function and expression of H7^a^ by normal host cells. Notably, mice in which anti-H7^a^ T cells did not persist long-term (day 100) nevertheless remained tumor-free. Thus, in this model, cure is probably due to eradication of clonogenic tumor cells rather than to induction of T-cell dependent tumor dormancy.

## Materials and Methods

### Mice, Tumor Cells and Statistics

We obtained B10.H7^b^(47N)/Sn (B10.H7^b^) and C57BL/10J (B10) mice from the Jackson Laboratory (Bar Harbor, ME) and the B16.F10 melanoma cell line from the American Type Culture Collection (Manassas, VA). Mouse care and experimental procedures were performed under approval from the Animal Care Committees of the University of Montreal and of the Maisonneuve-Rosemont Hospital. Mice were treated according to the guidelines of the Canadian Council on Animal Care. Differences between group means were tested using Student's *t* test.

### Cell Transplantation and Thymectomy

On day 0, H7^a^-positive (B10) and H7^a^-negative (B10.H7^b^) recipients received 12 Gy total-body irradiation, 10^7^ T cell-depleted bone marrow cells i.v. and 2×10^5^ tumor cells s.c. in the right flank. On day 7, recipients were treated with 5×10^7^ splenocytes from B10.H7^b^ donors primed against H7^a^. Priming of B10.H7^b^ donors against H7^a^ was performed by i.p. injection of 2×10^7^ B10 spleen cells 14 days prior to adoptive transfer. Tumor size was measured every 48 h and we sacrificed mice when the largest tumor diameter reached 17 mm. On day 100, we re-challenged cured mice with 2×10^5^ B16.F10 melanoma cells s.c. Thymectomy were performed as previously described [13].

### Cell Staining with Antibodies and Tetramers

We purchased antibodies specific for the following molecules: CD8 (53-6.7), from BD Pharmingen (San Jose, CA) and CD44 (11-0441), from eBioscience (San Diego, CA). We obtained phycoerythrin-labeled H7^a^-H2D^b^ tetramers from the tetramer core facility of the Canadian Network for Vaccines and Immunotherapeutics (Montreal, QC, Canada). Cells were stained as previously described and were analyzed on a FACSCalibur using the CellQuest program (BD Biosciences, San Jose, CA) [14], [15].

### Assessment of T-Cell Proliferative Dynamics from CFSE Profiles

We labeled splenocytes from B10.H7^b^ female mice (immunized on day −7 against H7^a^) with CFSE (Molecular Probes, Burlington, ON, Canada) as previously described [16], [17]. Briefly, splenocytes were suspended at a concentration of 5×10^7^ cells/ml in Hanks' balanced salt solution. After warming to 37°C, CFSE was added at a concentration of 5 µM for 15 minutes, followed by addition of ice-cold RPMI media and cell recovery by centrifugation. Donor cells were subsequently injected i.v. into the tail vein of recipient mice. Before analysis, we stained cell suspensions containing CFSE-labeled cells with anti-CD8 antibody and H7^a^-H2D^b^ tetramers. Division peaks (as determined by CFSE-intensity) were labeled from 0 to *n*. Since a single T cell dividing *n* times will generate 2*^n^* daughter cells, if the total number of T cells which have divided three times (*n* = 3) is eight, then exactly one precursor had to divide three times to generate these eight cells (2^3^ = 8) [17], [18]. Making use of this mathematical relationship, the number of T cells that have divided was extrapolated from the number of daughters under each division peak, and the total number of mitotic events was calculated as described [16], [19]. The proliferative burst size (number of daughter cells generated by a dividing T-cell “precursor”) was obtained by dividing the total number of mitoses by the number of precursors that had divided [19].

## Results

### Experimental Model (Fig. 1)

**Figure 1:**
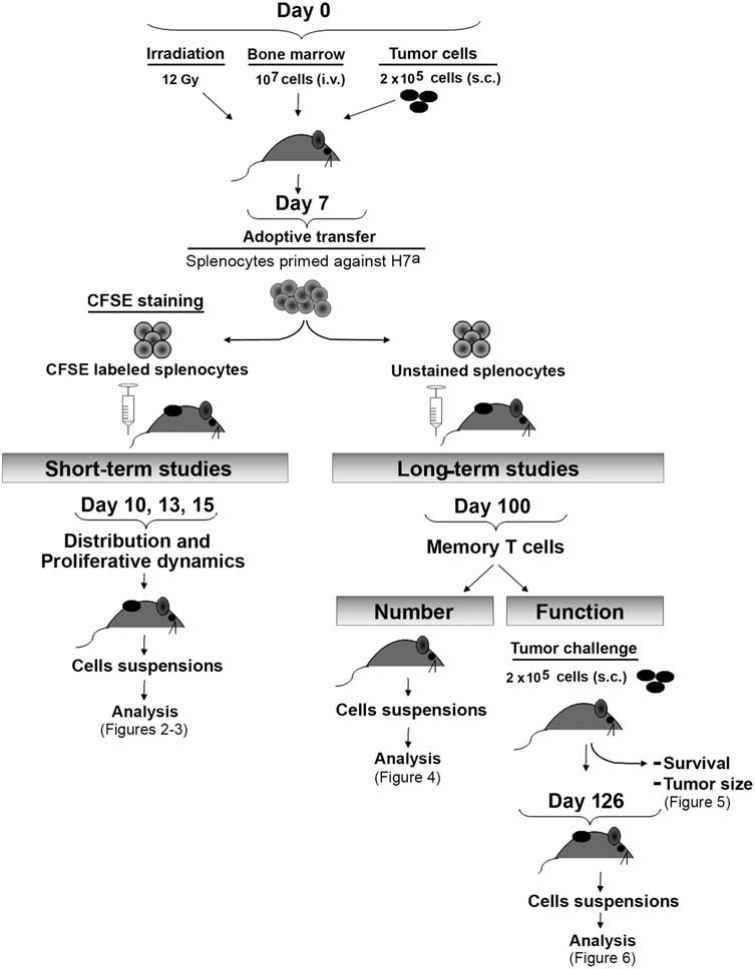
Experimental model and study design. Melanoma-bearing mice received splenocytes primed against H7^a^ on day 7. Short-term studies on T-cell proliferation kinetics were performed from day 10 to day 15, and long-term studies on memory T cells were initiated on day 100.

**Figure 2:**
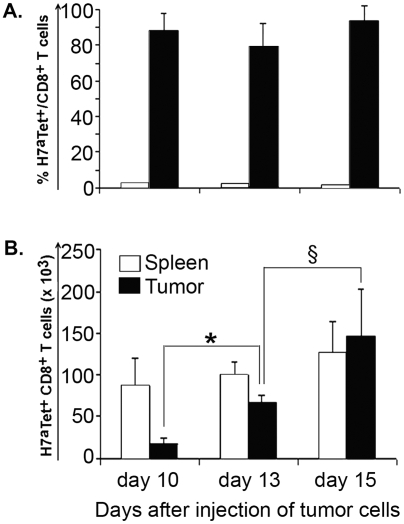
Anti-H7^a^ T cells accumulate in the tumor. We treated B10 recipient mice as described in Fig. 1. Cell suspensions from their spleen and tumor were obtained on day 10, 13 and 15, and stained with H7^a^ tetramers and anti-CD8 antibody. A) Proportion and B) absolute numbers of H7^a^ tetramer-positive CD8 T cells in 3–5 mice studied at each time point. * *P*<0.01; § *P*<0.001.

**Figure 3:**
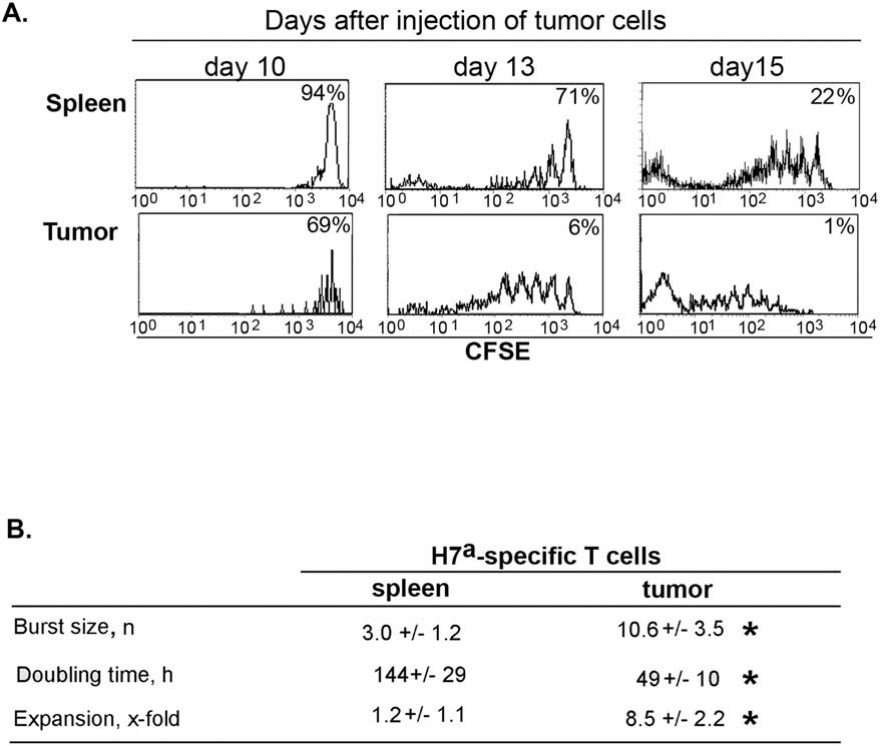
Evaluation of T-cell proliferative dynamics from CFSE profiles. B10 recipient were treated as in Fig. 1. A) One representative example of the CFSE profiles observed at each time point (gated on CD8^+^ H7^a^ tetramer^+^ T cells) in the spleen and tumor. Numbers in the upper right corner of each panel represent the percentage of undivided cells (that is, cells showing the highest levels of CFSE). B) Mean (±SD) burst size, doubling time and expansion of H7^a^-specific T cells between day 10 and day 15 in the spleen and tumor. The proliferative burst size and doubling time of anti-H7^a^ T cells were calculated as described in materials and methods. Expansion corresponds to the mean number of cells on day 15/mean number of cells on day 10. Each group contained 3–5 mice. * *P*<0.001 (spleen vs. tumor).

**Figure 4:**
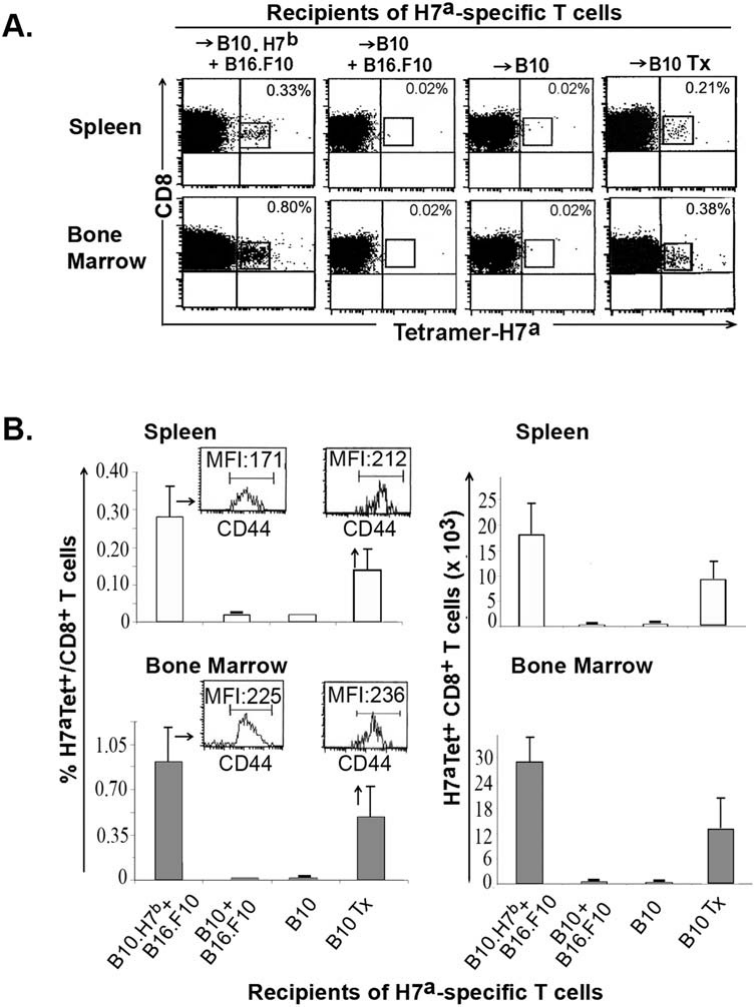
Persistence of H7^a^-specific CD8^+^ memory T cells. We studied four experimental groups: i) B10.H7^b^ and B10 mice successfully treated for melanoma as described in Fig. 1 (+B16.F10); ii) euthymic and thymectomized (Tx) B10 mice that underwent the same protocol excepting injection of melanoma cells on day 0. On day 100, we sacrificed 3 mice per group and stained cell suspensions from spleen and bone marrow with H7^a^ tetramers and antibodies against CD8 and CD44. A) Dot plots of one representative experiment out of three (gated on CD8 cells). B) Proportion and absolute number of H7^a^-specific T cells in recipients' spleen and bone marrow (2 tibiae and femurs). Histograms represent the mean±SD of 3 mice per group. Inserts depict CD44 expression on tetramer^+^ CD8 T cells found in euthymic B10.H7^b^ and thymectomized B10 hosts. MFI: mean fluorescence intensity.

**Figure 5:**
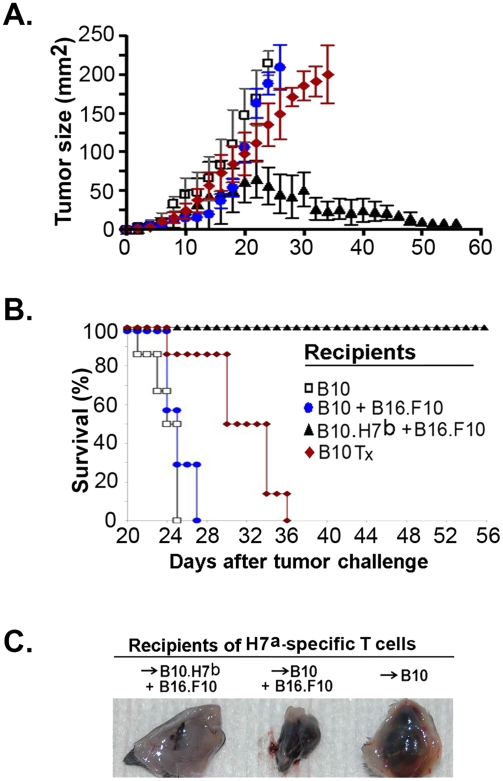
Challenge of long-term chimeras with melanoma cells. We studied mice from the four experimental groups described in Fig. 4. On day 100, mice were challenged with 2×10^5^ B16.F10 melanoma cells s.c. A) Tumor surface area and B) survival of experimental groups (n≥10 mice per group). The mean survival times for the following groups were significantly different: white vs. black, white vs. red, and blue vs. black, *P*<0.0001; red vs. black, *P*<0.001. C) Examination of excised tumor injection site shows large tumor nodules (black) in B10 mice but only an inconspicuous micro-nodule in the B10.H7^b^ mouse (one representative experiment out of 10). Tx, thymectomized.

**Figure 6:**
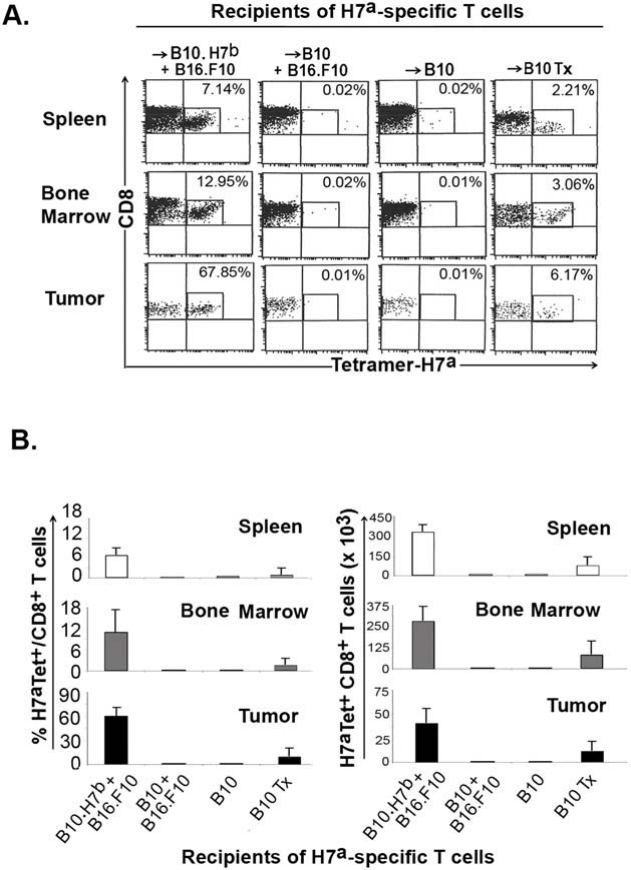
Anti-H7^a^ T-cell response following tumor challenge. We studied mice from the four experimental groups described in Fig. 4. On day 100, we challenged mice with 2×10^5^ B16.F10 melanoma cells s.c. Mice were sacrificed on day 126 and cell suspensions from their spleen, bone marrow and tumor were stained with H7^a^ tetramers and anti-CD8 antibody. A) Dot plots of one representative experiment out of three (gated on CD8 cells). B) Proportion and absolute number of H7^a^-specific T cells in recipients' spleen, bone marrow (2 tibiae and femurs) and tumor on day 126. Results are depicted as the mean±SD of 3 mice per group. Absolute numbers of H7^a^-specific T cells in the three sites were higher in thymectomized relative to euthymic B10 hosts (0.02<*P*<0.002), and were higher in B10.H7^b^ than in euthymic (*P*<0.0005) or thymectomized (*P*<0.01) B10 hosts. Tx, thymectomized.

On day 0, H7^a^-positive (B10) and H7^a^-negative (B10.H7^b^) mice received 12 Gy total-body irradiation, 10^7^ T cell-depleted bone marrow cells i.v. and 2×10^5^ B16.F10 melanoma cells (H7^a^-positive) s.c. On day 7, we treated mice by injection of 5×10^7^ splenocytes from B10.H7^b^ female immunized against H7^a^ 14 days prior to adoptive transfer. Splenocyte suspensions (5×10^7^ cells) from donors primed against H7^a^ contained 2.5±0.3×10^5^ H7^a^ tetramer-positive CD8 T cells (data not shown). We have previously reported the gene expression profile and cell surface phenotype H7^a^ tetramer-positive CD8 T cells primed under those conditions [7], [15]. CD8 T cells supplied with CD4 help at the time of initial priming generate more efficient effector cells upon secondary challenge [20], [21]. Thus, as in previous studies [6], [7], we used B10.H7^b^ female mice primed against B10 male cells as a source of anti-H7^a^ CD8 T cells. This immunization scheme leads to the expansion of anti-HY CD4 T cells and of anti-H7^a^ CD8 T cells in donor mice (immunodomination prevents expansion of anti-HY CD8 T cells) [22]–[24]. We used only female mice as recipients. Thus, following adoptive transfer only anti-H7^a^ CD8 T cells could encounter their cognate Ag in recipients because both recipients and tumor cells are HY-negative. Our model displays two features that make it relevant as a pre-clinical paradigm: i) our protocol involves a therapeutic rather than prophylactic setting [25]; ii) alike most spontaneous human tumors, B16.F10 cells do not express MHC II and display only low levels of MHC I molecules [26]. We displayed in Fig. 1 the overall study design and the timing of short-term and long-term studies whose results are presented in Fig. 2–3 and Fig. 4, 5, 6, respectively.

### Proliferation Kinetics of H7^a^-Specific CD8 T Cells

The outcome of adoptive immunotherapy is presumably dictated by the proliferative dynamics of both tumor cells and tumor infiltrating T cells. In the first set of experiments, we therefore sought to determine the rate of accumulation and proliferation of H7^a^-specific T cells in the tumor at early time points after adoptive transfer. To this end, anti-H7^a^ donor cells were stained with CFSE before injection in B10 recipients on day 7. We sacrificed mice on day 10, 13 and 15 (that is, day 3, 6 and 8 after adoptive transfer) and prepared cells suspensions from tumors and spleens to evaluate T-cell accumulation and mitotic behavior. Non-specific labeling with H7^a^ tetramers, as determined by staining naïve B10 or B10.H7^b^ CD8 T cells, was ≤0.04% (data not shown). At each time point, the proportion of CD8 T cells that were H7^a^ tetramer-positive was ≈1–2% in the spleen but ≈80–90% in the tumor (Fig. 2A). Thus, as early as day 10 (day 3 after adoptive transfer), we observed a selective accumulation of H7^a^-specific T cells in the tumors. Between day 10 and day 15, the absolute number of H7^a^-specific T cells remained relatively stable in the spleen but increased ≈8.5-fold in the tumor (Fig. 2B and 3B).

The expansion in the number of intratumoral H7^a^-specific CD8^+^ T cells could be due to their proliferation *in situ* or their recruitment from secondary lymphoid organs. To investigate this issue, we analyzed the CFSE content of H7^a^ tetramer-positive T cells present in the spleen and the tumor. The CFSE content decreases by 50% after each cell division. The vast majority of H7^a^-specific T cells harvested on day 10 had underwent 0 or 1 mitosis following adoptive transfer (Fig. 3A). From day 10 to 15, the mean CFSE content of anti-H7^a^ T cells decreased much more rapidly in the tumor than in the spleen (Fig. 3A). By day 15, all anti-H7^a^ T cells harvested from the tumor had divided at least once while a substantial number of anti-H7^a^ T cells in the spleen had not (Fig. 3A). Using equations developed by Turka *et al.* [17], [19] we calculated the proliferative burst size and the doubling time of H7^a^ tetramer-positive T cells (Fig. 3). The proliferative burst size corresponds to the number of daughter cells generated by a dividing T-cell “precursor”, and the doubling time represents the time required for the average T cell to achieve a single cell division. Our calculations are based on the assumption that H7^a^-specific T cells present in one site (spleen or tumor) on day 15 derive from H7^a^-specific T cells that had seeded this particular site by day 10. On day 15, H7^a^-specific T cells in the tumor displayed a greater burst size (≈3.5-fold) and shorter doubling time (≈3-fold) than H7^a^-specific T cells in the spleen (Fig. 3B).

Our studies on the proliferation kinetics of H7^a^-specific CD8 T cells show that H7^a^-specific T cells found in the tumor and spleen have different mitotic histories: H7^a^-specific T cells in the tumor have undergone more cell divisions that those in the spleen (Fig. 3). These data suggest that the massive expansion H7^a^-specific T cells found in the tumor, but not the spleen, reflects their intratumoral proliferation.

### Two Host Factors Regulate Long-Term Persistence of H7^a^-Specific Memory T Cells

In order to determine whether H7^a^-specific T cells persisted long-term following adoptive transfer, we assessed the numbers of H7^a^ tetramer^+^ CD8 T cells present on day 100 in the spleen and bone marrow of euthymic mice successfully treated for melanoma (as shown in Fig. 1). We paid special attention to the bone marrow because it is a preferential homing site for memory T cells, and memory CD8 T cells proliferate more extensively in the bone marrow than they do in either secondary lymphoid or extra-lymphoid organs [27]. Since H7^a^ expression by host cells might impinge on long-term persistence of H7^a^-specific T cells, we studied the fate of primed H7^a^-specific T cells transferred in B10 and B10.H7^b^ hosts. The salient finding was that significant numbers of H7^a^-specific T cells were found in B10.H7^b^ but not in B10 hosts (*P*<0.001; Fig. 4). The sole difference between these two strains of mice is that H7^a^ MiHA is ubiquitously expressed in B10 mice, but absent in B10.H7^b^ mice [6], [28], [29]. Thus, when confronted with ubiquitous expression of their cognate antigen, H7^a^-targeted T cells underwent apoptosis or replicative exhaustion. As expected for memory T cells, about 98% of anti-H7^a^ T cells were CD44^hi^. Consistent with the fact that the bone marrow is a preferential homing site for memory T cells [27], [30], the frequency of H7^a^-specific T cells was higher in the bone marrow than the spleen of B10.H7^b^ mice (*P*<0.01).

Naïve and memory CD8 T cells may compete for cytokines that regulate lymphocyte survival and proliferation [31]. We therefore asked whether in the absence of thymic output, anti-H7^a^ memory T cells would persist long-term in B10 hosts. To test this, we evaluated the persistence of H7^a^-specific memory T cells in euthymic vs. thymectomized H7^a^-positive (B10) hosts. Significant numbers of H7^a^-specific memory T cells were present on day 100 in thymectomized B10 hosts (*P*<0.01 relative to euthymic B10 hosts; Fig. 4). Thus, anti-H7^a^ T cells can persist long-term in B10 hosts in the absence of naïve T cell production by the thymus. We conclude that two host factors regulated persistence of anti-H7^a^ memory T cells: thymic function and expression of H7^a^ by normal host cells. H7^a^-specific memory T cells were abundant in euthymic B10.H7^b^ hosts, present in low numbers in thymectomized B10 hosts, and undetectable in euthymic B10 hosts.

### Challenge of Hematopoietic Chimeras with Tumor Cells

No melanoma recurrence was seen in B10 mice (n = 30) observed for 6 months after induction of complete remission by adoptive transfer of anti-H7^a^ T cells ([7] and data not shown). To test whether lack of tumor relapse might be due to persistence of H7^a^-specific T cells at levels below the detection limit of tetramer-staining assays, cured B10 recipients were challenged on day 100, with 2×10^5^ melanoma cells (as on day 0). In euthymic cured B10 recipients, tumors grew rapidly (Fig. 5) and no accumulation of H7^a^-specific T cells was detected in the spleen, bone marrow or tumor (Fig. 6). Thus, cured euthymic B10 mice did not show any functional evidence of immune reactivity to H7^a^ or to any tumor-associated epitopes. In thymectomized B10 hosts, a minimal accumulation of H7^a^-specific T cells was found (Fig. 6) that entailed only a small delay in tumor growth (Fig. 5). Hence, H7^a^-specific memory T cells present in thymectomized B10 hosts were unable to provide a protective antitumor response. In contrast, H7^a^-specific memory T cells present in B10.H7^b^ hosts generated a strong anamnestic response following tumor rechallenge (Fig. 6). In the latter mice, the tumor grew for a few days and then disappeared (Fig. 5). Thus, H7^a^-specific memory T cells found in B10.H7^b^ hosts were perfectly functional.

## Discussion

### Intratumoral Accumulation of H7^a^-Specific T Cells

A major obstacle encountered in cancer immunotherapy trials, including those targeting MiHAs, is the failure of antigen-reactive T cells to invade tumors and persist long-term [12], [32]–[34]. Few studies have addressed the *in vivo* fate of MiHA-specific T cells using MHC-peptide tetramers [35], [36]. Moreover, to the best of our knowledge, no study has evaluated the *in vivo* fate of antigen-primed MiHA-specific T cells, nor their behavior in the tumor environment. We previously reported that adoptively transferred anti-H7^a^ T cells were found in large numbers in regressing melanomas (day 19) [7]. Here, by looking at earlier time points, we found that selective tumor infiltration by anti-H7^a^ T cells was established by day 3 after adoptive transfer. Preferential localization of adoptively transferred CD8 T cells to tumor sites has been reported in one clinical trial [37]. Homing to the tumor is probably due to interaction between activated T cells and antigen-independent inflammatory ligands such as Vcam-1 [7], [38]. We found that following initial seeding, numbers of H7^a^-specific T cells increased dramatically in the tumor but not the spleen. Furthermore, based on CFSE profiles, we found that H7^a^-specific T cells in the tumor had divided much more extensively than those in the spleen. These data suggest that the massive accumulation of H7^a^-specific T cells in the tumor between day 10 and day 15 is due to *in situ* proliferation. They are also consistent with the fact that differentiation and survival of primed CD8 (but not CD4) effector T cells are independent of secondary lymphoid organs in adoptive hosts [39]. However, we cannot formally discard the possibility that, in our model, H7^a^-specific T cells proliferate in the draining lymph node and then migrate to the tumor. Further studies are needed to understand the nature of signals that drive intratumoral expansion of MiHA-specific T cells. We speculate that IFN-γ may be an important player in this process. Indeed, the local release of IFN-γ by anti-H7^a^ T cells upregulates expression of MHC I and H7^a^ in the tumor [7], and may thereby stimulate proliferation of anti-H7^a^ T cells. Nonetheless, though the tumor environment may not be particularly propitious to T-cell invasion and expansion [9]–[12], the present work illustrates that this limitation may be overcome by adoptive transfer of primed CD8 T cells targeted to an immunodominant MiHA. In line with this, new promising methods have been recently developed to generate high avidity MiHA-specific CD8 T cells for adoptive immunotherapy in human [40]–[42].

### Relapse-Free Survival Did Not Necessitate Persistence of H7^a^-Reactive Memory T Cells

Whether long-term cancer remission induced by MiHA-targeted T cells is associated with total eradication of cancer cells or with persistence of low numbers of resting or slow growing tumor cells (tumor dormancy) is unknown. The distinction between these two outcomes is important since tumor persistence always entails the risk of relapse. Genuine cancer cure requires elimination of cancer stem cells which have a mostly quiescent cell cycle profile and are capable of self-renewal. The quiescent status of cancer stem cells renders them difficult to eradicate with chemotherapy that typically target proliferating cells. Nonetheless, T cells can eliminate quiescent cells as well as cycling cells. Thus, when cultured with acute myeloid leukemia cells, MiHA-specific T cells can wipe out leukemia stem cells [43]. Nevertheless, a substantial body of evidence suggests that remissions induced by various types of immunotherapy targeted to tumor specific antigens (but not to MiHAs) are usually associated with tumor dormancy [44] We found that recipients in which anti-H7^a^ T cells disappeared (euthymic B10 mice) nevertheless remained tumor-free. The latter mice behave as naive mice when rechallenged with melanoma cells. Thus, in this model, cure is probably due to eradication of clonogenic tumor cells rather than to induction of T-cell dependent tumor dormancy. Notably, we have observed no late relapses in mice with EL4 leukemia treated with anti-H7^a^ T cells ([6] and unpublished observations). This suggests that the findings reported herein may not be unique to melanoma and may be relevant to some rapidly growing tumors like B16.F10 and EL4. By no means, however, do we infer that persistence of memory T cells is generally irrelevant in adoptive cancer immunotherapy. The occurrence of late leukemia relapses following allogeneic hematopoietic cell transplantation argues against this view. Nevertheless, the lack of cancer relapse in mice devoid of anti-H7^a^ memory T cells illustrates the potency of the acute anti-tumor effect that can be generated by anti-MiHA T cells.

### Antigen Distribution and Thymus Function Regulate Long-Term Persistence of H7^a^-Specific Memory T Cells

Functional anti-H7^a^ T cells persisted long-term in B10.H7^b^ but not B10 mice. The ubiquitous expression of H7^a^ on B10 host cells led to physical demise or functional impairment of anti-H7^a^ T cells. The exhaustion of H7^a^-specific T cells in B10 recipients was similar to that of anti-viral T cells confronted with viruses that disseminate widely [45]. Thus, at least in euthymic subjects, adoptively transferred host-reactive T cells should have a longer life span when targeted to tissue-restricted (e.g., tumor-specific) as opposed to ubiquitous epitopes. Assuming that prolonged persistence of adoptively transferred T cells is probably relevant in preventing late cancer relapses, it would therefore be advantageous to target MiHAs with a restricted tissue distribution. Fortunately, several non ubiquitous MiHAs have been discovered in human [46]–[48]. Accordingly, MiHAs derived from oncoproteins, such as HA-1, represent particularly attractive targets for adoptive immunotherapy of hematopoietic malignancies and solid tumors [49], [50]. Finally, we found that thymic output had a negative impact on persistence of H7^a^-specific memory T cells. Many cancer patients, particularly those in older age groups, present thymic insufficiency [51]. MiHA-specific T cells may have a more protracted survival in these subjects. From a different perspective, the concept that thymic output mitigates the persistence of MiHA-reactive T cells could explain why graft-versus-host disease becomes more frequent with increasing recipient age following conventional allogeneic hematopoietic cell transplantation [52], [53].
